# Multi-modality curative treatment of salivary gland cancer liver metastases with drug-eluting bead chemoembolization, radiofrequency ablation, and surgical resection: a case report

**DOI:** 10.1186/1752-1947-5-416

**Published:** 2011-08-25

**Authors:** Andreas Karatzas, Konstantinos Katsanos, Ioannis Maroulis, Christina Kalogeropoulou, Evaggelos Tzorakoleftherakis, Dimitris Karnabatidis

**Affiliations:** 1 Department of Radiology, University Hospital of Patras, Rion, Patras 26504, Greece; 2 Department of Interventional Radiology, University Hospital of Patras, Rion, Patras 26504, Greece; 3 Department of Surgery, University Hospital of Patras, Rion, Patras 26504, Greece

## Abstract

**Introduction:**

Liver metastases are rare in salivary gland tumors and have been reported only once to be the first manifestation of the disease. They are usually treated with surgical resection of the primary tumor and systemic chemotherapy. Drug-eluting bead chemoembolization has an evolving role in the treatment of hepatocellular carcinoma, as well as in the treatment of metastatic disease of the liver. Nevertheless, it has never been used in a patient with salivary gland liver metastases.

**Case presentation:**

We report a case of a 51-year-old Caucasian Greek woman who presented to our hospital with liver metastases as the first manifestation of an adenoid cystic carcinoma of the left submandibular gland. The liver lesions were deemed inoperable because of their size and multi-focality and proved resistant to systemic chemotherapy. She was curatively treated with a combination of doxorubicin eluting bead (DC Beads) chemoembolization, intra-operative and percutaneous radiofrequency ablation, and radiofrequency-assisted surgical resection. The patient remained disease-free one year after the surgical resection.

**Conclusion:**

In conclusion, this complex case is an example of inoperable liver metastatic disease from the salivary glands that was refractory to systemic chemotherapy but was curatively treated with a combination of locoregional therapies and surgery. A multi-disciplinary approach and the adoption of modern radiological techniques produced good results after conventional therapies failed and there were no other available treatment modalities.

## Introduction

Salivary gland tumors are rare, histologically diverse, and exhibit a variety of biological behaviors. They account for 11% of all oropharyngeal neoplasms, with an incidence of 11.95/1,000,000/year in the United States [1]. The frequency of distal metastases varies according to the histological type [2,3]. Liver metastases are overall quite rare and occur in advanced cases. It is extremely rare for liver metastases to be the initial presentation, and, to the best of our knowledge, only one case has been reported in the literature [4].

Treatment of primary and secondary liver tumors has changed during the past few years. New techniques, both invasive and minimally invasive surgery, have emerged and offer potential cure to patients who in the past would have been considered candidates only for palliative treatment. These treatments include drug-eluting bead (DEB) chemoembolization, percutaneous and intra-operative ablation, and atypical surgical resection techniques. There is no report in the literature on the use of DEB chemoembolization for this type of metastatic tumor.

We report a rare case of a patient with salivary gland cancer who presented to our hospital with initially inoperable advanced metastatic liver disease as the first manifestation of the disease. The patient was first treated successfully with a combination of locoregional treatments and then underwent curative surgical resection.

## Case presentation

A 51-year-old Caucasian Greek woman had a routine liver ultrasound examination following a history of large-bowel polypectomies. Multiple hypoechoic liver lesions were found, the largest of which, on segment VI, measured 2.2 cm. The patient was asymptomatic, and her physical examination was unremarkable. Routine laboratory examinations showed no abnormal findings. Apart from her history of large-bowel polypectomies, she only had a history of appendectomy 35 years ago and bilateral saphenectomy 23 years ago. To avoid exposing the patient to ionizing radiation, the referring physician decided to further evaluate the lesions with MRI of the abdomen, which demonstrated a total of eight liver lesions in both lobes. The lesions had reduced signaling on T1-weighted sequences and increased signaling on T2-weighted sequences. They did not show enhancement in the arterial phase and had reduced enhancement compared to the normal liver parenchyma in the portal venous phase. On the basis of these features, metastatic liver disease was considered as the first-line diagnosis. MRI did not show any other abnormal findings.

**Figure 1:**
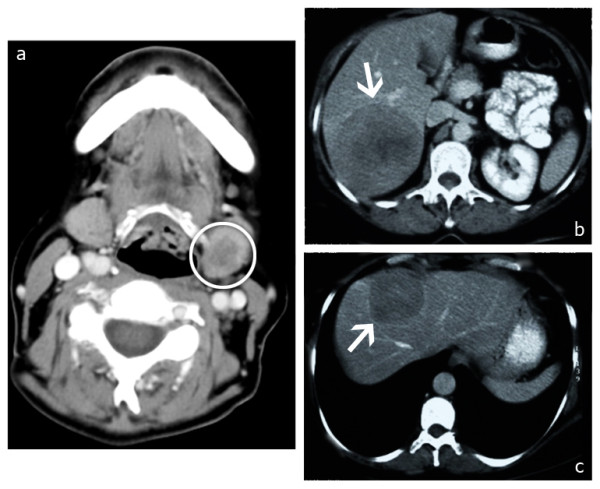
**Pre-therapy imaging**. **(a) **The primary tumor. Adenoid cystic carcinoma of the left submandibular salivary gland is indicated by the circle. **(b) **and **(c) **Computed tomography (CT) of the liver before chemoembolization showing the two main lesions. **(b) **A 10 cm lesion involving segments VI and VII is indicated by the arrow. **(c) **A 4.7 cm lesion involving segments VIII and IV_A _is indicated by the arrow. Three more lesions in segments IV_A_, IV_B_, and II, which measured 1.5 cm, 1 cm, and 1.8 cm, respectively, were identified.

To reveal the primary lesion, the patient was further investigated with gastroscopy, colonoscopy, and computed tomography (CT) of the head, lung, and abdomen, all of which were unremarkable. Fine-needle aspiration under CT guidance was performed on the lesion in segment VI. The cytology was positive for malignancy, the type of which, though, could not be defined. Diagnostic laparoscopy was then performed, and the anterior superficial lesion in segment II was resected. The biopsy specimen submitted for pathological examination showed metastatic cancer with features of primary disease from a cystic adenoid carcinoma of the salivary gland. Neck CT was performed, which revealed a 1.5 cm hypodense area on the left submandibular gland (Figure 1), thus confirming the diagnosis.

The left submandibular gland was removed with clear surgical margins, and the patient was started on chemotherapy treatment with cisplatin, doxorubicin, and epirubicin. Three months later a new liver MRI scan showed disease progression, and the chemotherapy was switched to a combination of 5-fluorouracil (5-FU), cisplatin, and cyclophosphamide. After 3 more months, MRI demonstrated further enlargement of the liver lesions, with the diameter of the biggest of them measuring 10 cm (Figure 1).

**Figure 2:**
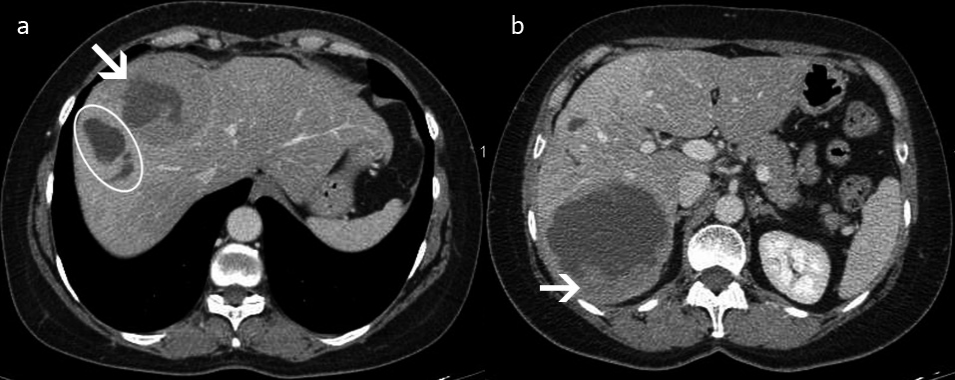
**CT of the liver after chemoembolization**. Two partially necrotic lesions remained. The first, 8.9 cm in diameter, extended into segments VI and VII, and the second, 4.2 cm in diameter, extended into segments VIII and IV_A_. **(a) **Arrow indicates the necrosis within the lesion. The circle indicates necrosis of the normal liver parenchyma adjacent to the lesion in segment VIII. **(b) **The viable tumor at the periphery of the lesion is shown.

At that time, chemoembolization with DC beads loaded with doxorubicin was considered the best therapeutic option. Two sessions were performed two months apart, and 150 mg of doxorubicin, loaded into DC beads 300 mm to 500 mm and 500 mm to 700 mm in diameter were injected each time. Because of the size of the lesions and their bilateral location, the chemoembolization was performed from the distal part of the right and left hepatic arteries. The patient had post-embolization syndrome, mainly with pain at the right upper quadrant, which was worse after the second embolization and was relieved only with opioid analgesics. No major complications were noted. Partial necrosis of the two larger lesions and complete obliteration of the three smaller ones were noted on CT scans one month after the first chemoembolization, and a further response was demonstrated on CT scans one month after the second chemoembolization (Figure 2).

**Figure 3:**
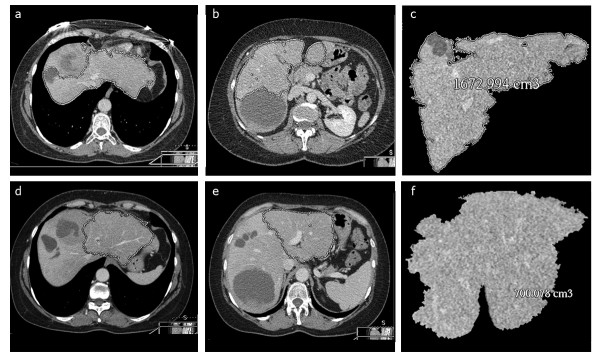
**CT volumetry of the liver**. **(d) **through **(f) **The volume of segments II, III, and IV_B_, which were going to be spared during surgery, were calculated and **(a) **through **(c) **divided by the volume of the functional liver before surgery. **(a) **and **(b) **The lesions were excluded for the calculation of the functional liver volume.

Taking into consideration the partial response according to the European Association for the Study of the Liver criteria [5] and the left liver lobe hypertrophy in the interim, surgical resection of the right liver and segment IV_A _was discussed and CT volumetry was performed (Figure 3). The estimated functional liver remnant was calculated as 41.8%, and the decision to perform surgery was made.

**Figure 4:**
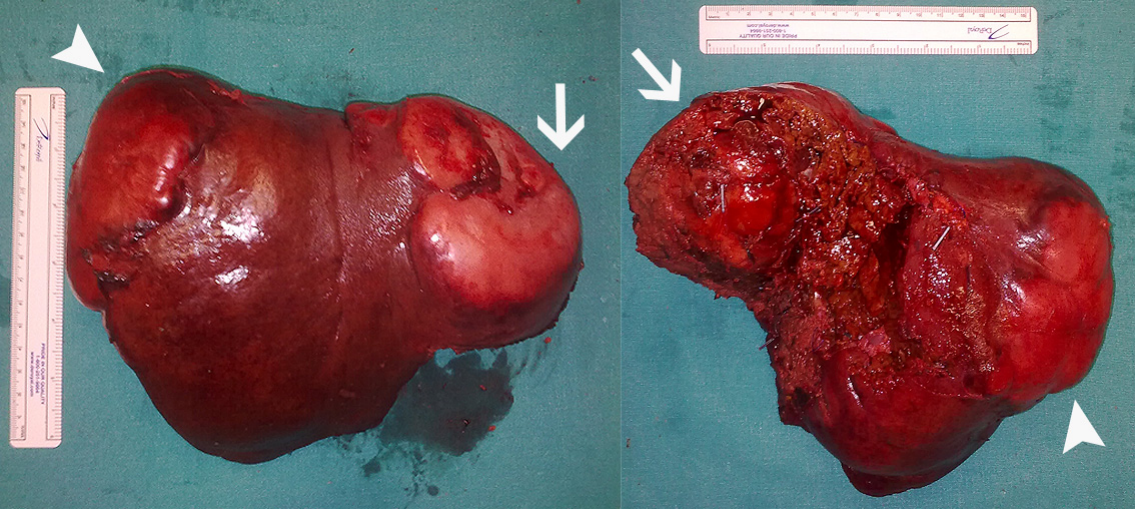
**Specimen of the resected liver**. The tumors in segments VI and VII (arrowheads) and segments VIII and IV_A _(arrows) are shown.

An atypical extended right hepatectomy was performed, and the right liver lobe, along with segment IV_A _of the liver, was resected using a RF-assisted technique to minimize bleeding complications (Figure 4). Intra-operative ultrasound revealed one more lesion on segment II measuring 0.9 cm, which was treated with intra-operative RF ablation. The surgical margins of the removed liver were found to be disease-free according to the pathology report.

The post-operative recovery of the patient was uneventful apart from a small right pleural effusion, which did not require further treatment. There was no liver dysfunction aside from transient elevation of total bilirubin up to 7.8 mg/dL (direct bilirubin 4.7 mg/dL). The patient was discharged 15 days after surgery in good general condition.

**Figure 5:**
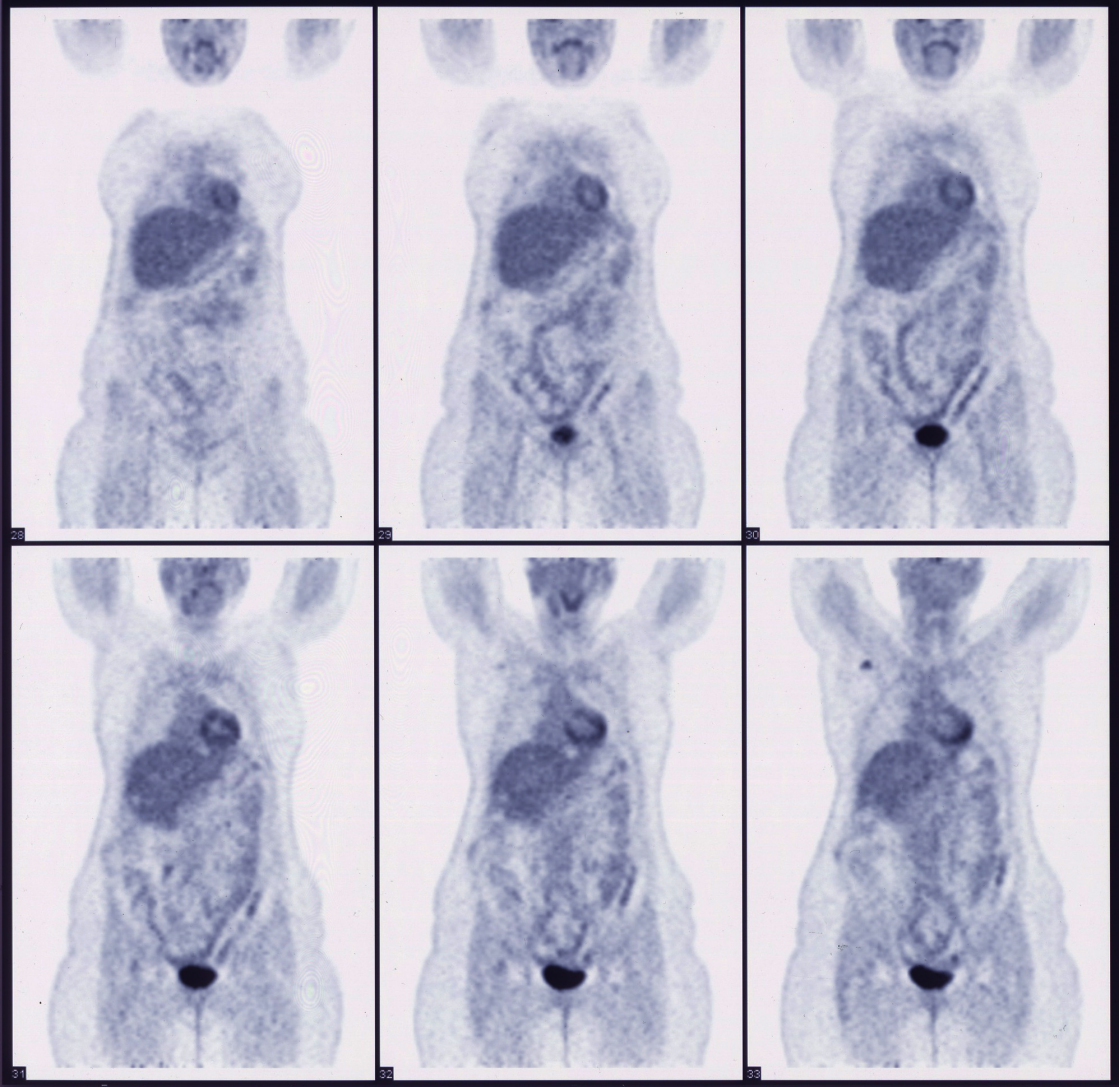
**Positron emission tomography scan at one-year follow-up**. The PET scan was negative for any recurrent disease.

Follow-up CT three months after surgery showed a new lesion measuring less than 1 cm on segment III. This was treated with a single session of percutaneous RF ablation. Eight months after surgery CT of the neck, chest, abdomen, and pelvis showed that the patient was free of disease. At the one-year post-operative follow-up examination, a positron emission tomography (PET) scan (Figure 5) and MRI of the abdomen showed that the patient remained free of any metastatic disease.

## Discussion

Liver metastasis from salivary gland cancer is an uncommon manifestation of the disease, so evidence is scarce concerning the appropriate treatment. Adenoid cystic carcinoma, contrary to most other salivary gland tumors, is an aggressive tumor with a relatively high ratio of distant metastases. Patients with distant metastases have been shown to have a three-year survival rate of 41% to 46% [6,7]. Surgical resection of the primary tumor and systemic chemotherapy are recommended [8,9], although surgical resection of solitary metastases has been reported [10]. The chemotherapeutic agents that have shown the best results as monotherapy are cisplatin, 5-FU, and doxorubicin. The combination of cyclophosphamide plus doxorubicin and cisplatin has proven to be the most effective chemotherapy treatment [7].

Chemoembolization has been widely used for liver metastatic disease, mainly for colorectal and neuroendocrine tumor metastases [11]. Drug-eluting microspheres have been introduced recently and have been used for the treatment of hepatocellular carcinoma and metastatic disease. It has been shown that they have more favorable pharmacokinetics than conventional chemoembolization, since they provide higher concentrations of the chemotherapeutic agent in the area of the tumor and less in the systemic circulation. Additionally, the chemotherapeutic agent remains in the area of the tumor for a longer period [12,13]. This case shows the potential of chemoembolization with DC Beads for the treatment of various metastatic tumors, even those with relatively low vascularity.

Bearing in mind the chemotherapeutic agents to which the patient showed no response when treated systemically, as well as the regimens proposed in the literature for metastatic salivary gland tumors, we decided that doxorubicin was the best choice. The ability to load it into DC Beads was another advantage that could not be overlooked. Because of the size of the lesion, the chemoembolization could not be selective. Given also the fact that the liver of the patient was not cirrhotic, both lobes were treated each time.

The good response of the patient to the first two chemoembolizations posed a therapeutic dilemma about the next step. Our goal was to offer surgery to the patient as a potentially curative treatment. The minor down-sizing of the lesions, along with the left lobe hypertrophy, allowed us to proceed with surgery. Resection of the tumor with a remaining functional liver volume greater than 20% is considered to be safe for a non-cirrhotic liver. In our patient, the estimated relative liver remnant was calculated to be 41.8%. This would also allow the safe intra-operative ablation of any remaining foci in the left lobe. Portal vein embolization was also considered before starting the chemoembolization as an alternative treatment that could produce left lobe hypertrophy and potentially offer the opportunity to perform surgery. However, this option was rejected because of the rapid progression of the lesions.

During surgery, intra-operative ultrasound revealed one more lesion that was not visible on CT scans. It has been shown that intra-operative ultrasound is a very sensitive modality for the detection of small metastases in the liver [14] and is considered a mandatory part of the procedure [15]. Nevertheless, there was still concern that additional microscopic foci could exist. This warranted prompt follow-up, which indeed was successful in revealing an early recurrence small enough to be treated with percutaneous RF ablation. The modality and frequency of follow-up for such a metastatic tumor is not well established. We used CT at three months and then at eight months because CT of the liver was also performed during the percutaneous RF ablation. At the patient's one-year follow-up examination, we used MRI of the abdomen and PET in an effort to reduce the radiation dose, bearing in mind, though, that since there was no previous PET scan, the tumor could be PET-negative and render a false-negative result. In this way, we managed to reduce the effective dose from 25mSv (CT of the neck, chest, abdomen, and pelvis) to approximately 14mSv (PET scan with low-dose CT).

Surgery is definitely not the treatment of choice for patients with metastatic liver disease from an adenoid cystic carcinoma, since even without treatment survival can be prolonged and there is no solid evidence that resection of the metastases prolongs survival. However, the biologic behavior of the tumor of this particular patient, bearing in mind the rapid increase in the size of the lesions, was quite aggressive. Furthermore, the age of the patient rendered her a good candidate for surgery, and she urged us to offer a more aggressive therapeutic approach to offer her a chance of living disease-free and possibly prolonging her survival. Nevertheless, further follow-up is needed, since late recurrence occurs quite often in patients with adenoid cystic carcinoma, but since the tumor burden in our patient was reduced, any recurrence would be more easily treatable with RF ablation.

## Conclusion

It is important to highlight the synergistic role of modern diagnostic and interventional techniques to the outcome of this case. Chemoembolization with DC Beads hindered the progression of the disease and achieved a partial response. Thin-section CT volumetry contributed to the decision to perform surgery by accurately calculating the functional liver remnant. Intra-operative ultrasound revealed a lesion that had not been pinpointed by conventional imaging techniques and also provided real-time guidance for the intra-operative ablation. Finally, percutaneous RF ablation permitted treatment of the disease recurrence with minimal invasion.

In conclusion, this case is a rare manifestation of adenoid cystic carcinoma of the salivary glands. Additionally, it is an example of inoperable liver metastatic disease refractory to systemic chemotherapy which was curatively treated with a combination of locoregional therapies and surgery. A multi-disciplinary approach and the adoption of modern radiological techniques produced good results after conventional therapies had failed and there were no other available therapeutic modalities.

## Consent

Written informed consent was obtained from the patient for publication of this case report and any accompanying images. A copy of the written consent is available for review by the Editor-in-Chief of this journal.

## Competing interests

The authors declare that they have no competing interests.

## Authors' contributions

AK participated in the interventional radiology procedures and the review of the imaging examinations and was a major contributor in the writing of the manuscript. KK participated in the interventional radiology procedures, performed the intra-operative ultrasound, and was a major contributor in the revision of the manuscript. IM performed the surgery and contributed to the surgical aspects of the manuscript. CK reviewed the imaging examinations and revised the manuscript. ET performed the surgery and revised the manuscript. DK participated in the interventional radiology procedures and made the final changes to the manuscript. All authors participated in the decision making of this case. All authors read and approved the final manuscript.
